# Inhibitory effect of piperine on *Helicobacter pylori* growth and adhesion to gastric adenocarcinoma cells

**DOI:** 10.1186/1750-9378-9-43

**Published:** 2014-12-16

**Authors:** Nagendran Tharmalingam, Sa-Hyun Kim, Min Park, Hyun Jun Woo, Hyun Woo Kim, Ji Yeong Yang, Ki-Jong Rhee, Jong Bae Kim

**Affiliations:** Department of Biomedical Laboratory Science, College of Health Sciences, Yonsei University, Wonju, Republic of Korea; Department of Clinical Laboratory Science, Semyung University, Jaecheon, Republic of Korea

**Keywords:** Piperine, *Helicobacter pylori*, Adhesion, Gastric cancer

## Abstract

**Background:**

Piperine is a compound comprising 5-9% of black pepper (*Piper nigrum*), which has a variety of biological roles related to anticancer activities. *Helicobacter pylori* has been classified as a gastric carcinogen, because it causes gastritis and gastric cancer by injecting the virulent toxin CagA and translocating VacA. The present study investigated the inhibitory action of piperine on *H. pylori* growth and adhesion.

**Methods:**

Inhibition of *H. pylori* growth was determined by the broth macrodilution method, and adhesion to gastric adenocarcinoma cells validated by urease assay. Motility test was performed by motility agar and the expression of adhesion gene and flagellar gene in response to the piperine treatment was assessed by RT-PCR and immunoblotting.

**Results:**

Administrated piperine suppressed the level of *H. pylori* adhesion to gastric adenocarcinoma cells in a dose dependent manner and the inhibition was statistically significant as determined by Student’s *t*-test. In addition, piperine treatment effects on the flagellar hook gene *flgE* and integral membrane component of the export apparatus gene *flhA* expression to be suppressed and piperine diminished the *H. pylori* motility.

**Conclusions:**

*flhA*, encodes an integral membrane component of the export apparatus, which is also one of the regulatory protein in the class 2 genes expression and *flgE* is one of them that encodes hook part of the flagella. Suppression of both genes, leads to less motility results in the organism attracted less towards to the gastric epithelial cells might be the possible reason in the adhesion inhibition. To our knowledge, this is the first report published on the inhibitory effects of piperine against the adhesion of *H. pylori* to gastric adenocarcinoma cells.

## Background

*Helicobacter pylori* is a gastric organism known for its association with chronic gastritis and peptic ulcers [1], as well as the development of gastric cancer [2]. Approximately half of the world’s population harbors this gastric pathogen. According to the World Health Organization, the organism causes gastric cancer and has been classified as a class I carcinogen [3]. Adhesion to gastric epithelial cells is the initial step in *H. pylori* infection, and this organism express at least six different adhesion-associated factors; AlpA-B (adherence-associated lipoprotein A and B), BabA (blood group antigen-binding adhesion), SabA (sialic acid-binding adhesion), HopZ (*H. pylori* outer membrane protein), HpaA (*H. pylori* adhesin A) and Lewis^x^-LPS, all of which mediate adhesion to gastric epithelial cells, followed by the establishment of infection [4]. Motility plays a vital role in colonization also it enhances *H. pylori* adhesion, and the motility is mediated by the sheathed flagella [5]. *H. pylori* flagellin is encoded by *flaA* and *flaB* to form filament, which is the part of flagella. This filament connects to the hook encoded by *flgE. flhA* encodes an integral membrane component of the export apparatus and regulates the expression of FlaA and FlaB [6]. Once an infection has commenced, the CagA protein is injected into epithelial cells via type IV secretion system [7] which then initiates multistep carcinogenesis [8].

Natural compounds or food supplements are now considered to be important substances studied for their anticancer activities during initiation, development and progression of cancer. Black pepper (*Piper nigrum*) is known as the “king of spices” and has been reported to have beneficial effects on common colds, coughs, dyspnea, throat diseases, intermittent fevers, colic, and dysentery. Traditionally, it has been used to treat inflammation [9]. Piperine, a nitrogenous substance that is abundantly present in black pepper, exhibits various roles in lipid and drug metabolism, bioavailability of drugs, and expresses antimutagenic and tumor-inhibiting effects as an antioxidant with influence on the gastrointestinal system [10]. Several researchers have reported that piperine has a role in anticancer activity as studied in various cancer cell lines, such as the inhibition of lung metastasis [11] and inhibition of prostate cancer [12]. In this study, we observed that piperine has a potential role in the growth inhibition of *H. pylori* and based on this observation, we hypothesized that, if piperine inhibits bacterial growth, it may also have a role in protecting against bacterial infection. Adhesion is the initial step in *H. pylori* infection and we used this factor as the rationale of our study. Our results demonstrated that piperine actively inhibits *H. pylori* adhesion, which was confirmed by urease assay and RT- PCR, immunoblotting and motility by motility agar test. To the best of our knowledge, this is the first report of piperine actively inhibiting *H. pylori* growth and adhesion.

## Results

### Effect of piperine on growth inhibition

**Figure 1:**
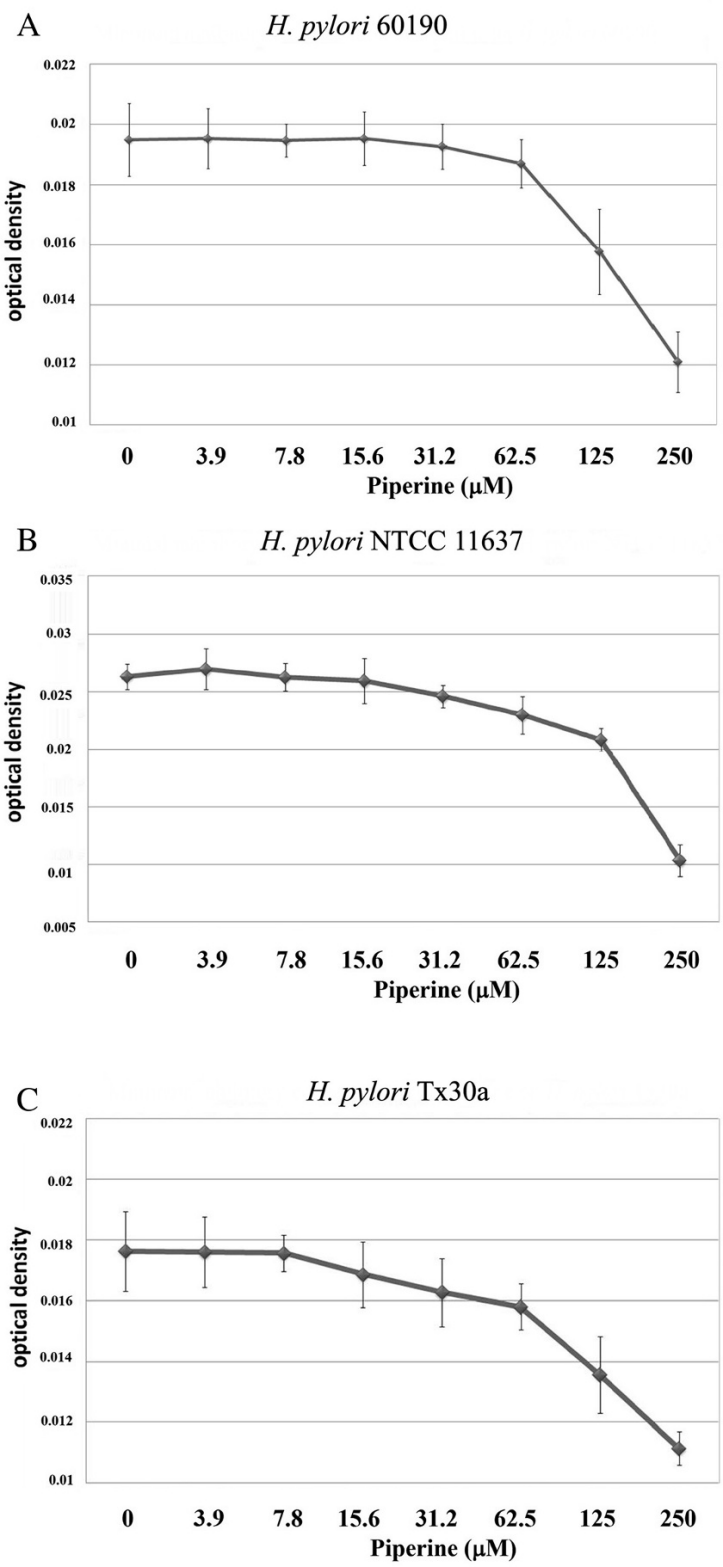
**Growth inhibition of piperine on** ***H. pylori*** **strains. A)** 60190; **B)** NCTC 11637; **C)** Tx30a. The growth inhibition was determined by the broth macrodilution method. Piperine concentrations from 3.9 μM to 250 μM were used in this growth inhibition assay compared to DMSO-treated control. Growth inhibition is expressed as optical density and 125 μM concentration of piperine shows minimal inhibitory concentration on *H. pylori* growth. The image is representative of three independent experiments and bars represent means ± SEM.

*H. pylori* strains 60190, NTCC 11637 and Tx30a were used in this study to determine the inhibitory action of piperine against *H. pylori* growth. The minimum concentration required to inhibit the complete growth of the *H. pylori* was found to be 125 μM (Figure 1A - C) determined by the broth macrodilution method. To confirm this finding, the agar dilution method was performed and the minimal inhibitory concentration of piperine on *H. pylori* growth was found to be same with broth macrodilution method (data not shown). For further experiments, 100 μM of piperine was administered at a sub-MIC concentration.

### Half maximal inhibitory concentration (IC_50_)

**Figure 2:**
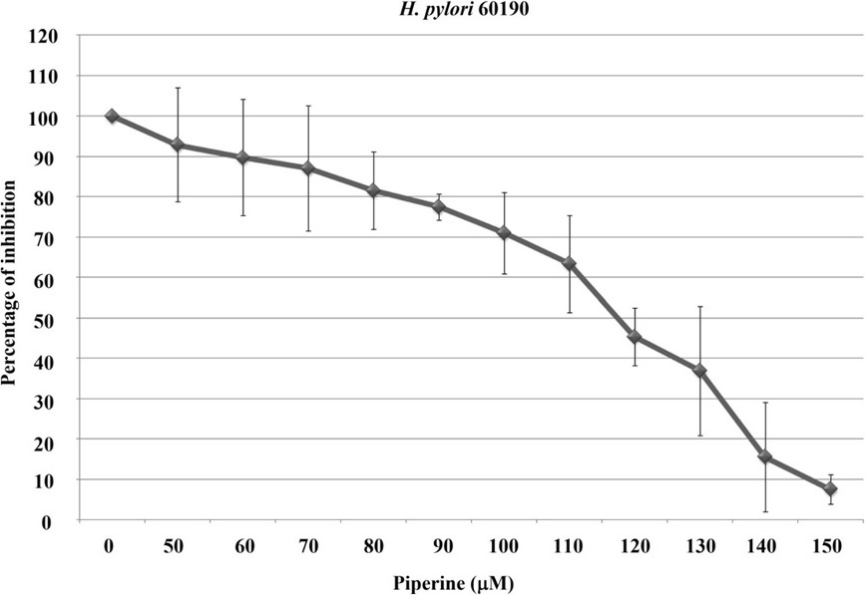
**Half maximal inhibitory concentration (IC**_**50**_ **).** IC_50_ value was determined by the broth culture method. Piperine concentrations of 50, 60, 70, 80, 90, 100, 110, 120, 130, 140, 150 μM were used and compared to DMSO treated control. IC_50_ value was determined as 115 μM by percentage of inhibition was plotted on nonlinear regression from logarithmic concentrations of piperine versus normalized response with variable slope. The image is representative of three independent experiments and bars represent means ± SEM.

*H. pylori* 60190 was used in this study to determine IC_50_ of the piperine which is defined as a measure of effectiveness of a compound in inhibiting biological or biochemical function. The quantitative measure indicates concentration of inhibiting compound required for given biological or biochemical function by half. *H. pylori* was treated with piperine at 50 ~ 150 μΜ. As shown in Figure 2 (Figure 2) the dose dependent decrease in the bacterial growth by piperine was expressed as a percentage and the IC_50_ value was found to be 115 μΜ from log concentrations of piperine in normalized response with variable slope.

### Effect of piperine on adhesion inhibition

**Figure 3:**
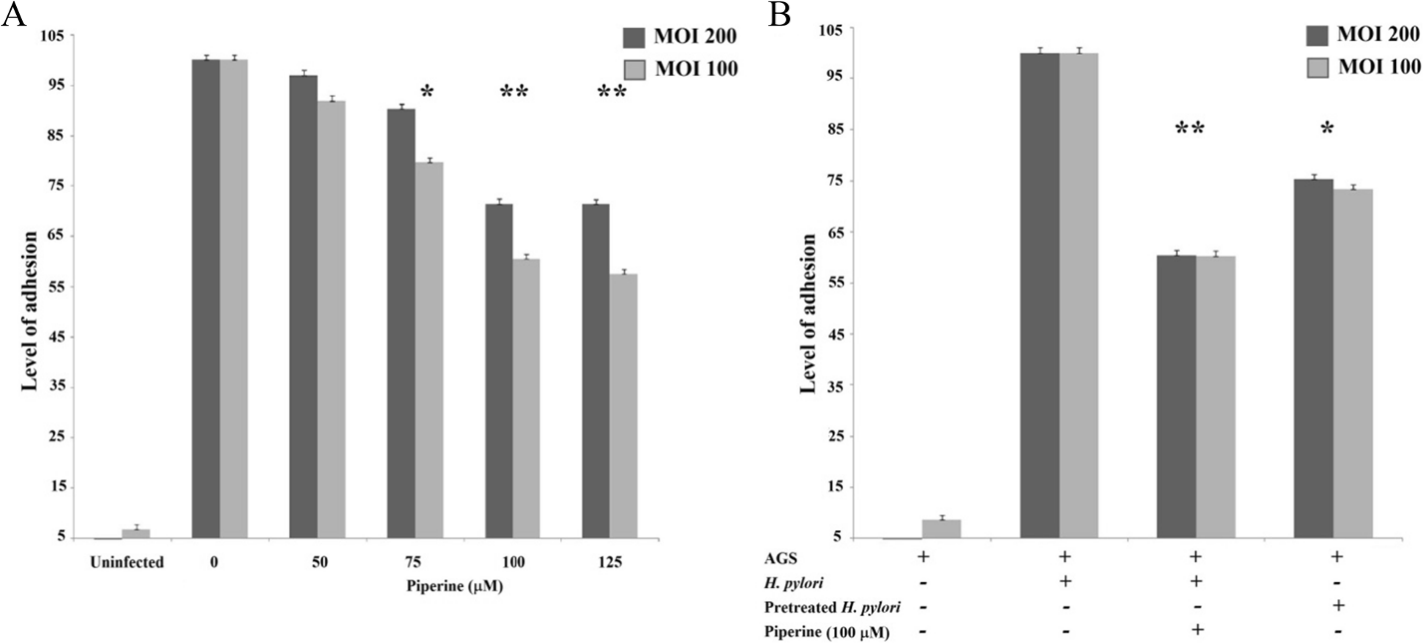
**Adhesion inhibition of piperine on** ***H. pylori*** **60190. A)** The inhibition of *H. pylori* adhesion to gastric adenocarcinoma cells by various concentrations of piperine; **B)** The inhibition of *H. pylori* 60190 adhesion to gastric adenocarcinoma cells by 100 μM of piperine or *H. pylori* pretreated with 100 μM of piperine for 24 hours. The level of adhesion was determined by urease test and the piperine-treated samples were compared to the DMSO-treated control. *H. pylori* 60190 adhesion to gastric adenocarcinoma cells was inhibited by piperine in a dose dependent manner. The image is representative of three independent experiments and bars represent means ± SEM. *p <0.05, **p <0.01.

*H. pylori* strain 60190 was used to determine whether piperine could inhibit adhesion of *H. pylori* to gastric epithelial cells. Piperine treatment at concentrations of 50, 75, 100 and 125 μM were applied during *H. pylori* infection for 2 hours, which reduced the adhesion of *H. pylori* to the AGS cells. Concentrations of 125 and 100 μM showed the least adhesion (Figure 3A) and potential bacterial adhesion inhibition was statistically highly significant (p < 0.01). At a concentration of 75 μM, significant inhibition (p < 0.05) was observed, but 50 μM did not appear to cause significant inhibition (Figure 3A). The results were similar at both 100 and 200 MOI and the adhesion of *H. pylori* to AGS cells was inversely proportional to the amount of piperine treatment. In summary, bacterial adhesion was reduced by piperine in a dose-dependent manner.

The minimum inhibitory concentration of piperine on *H. pylori* was found to be 125 μM and therefore 100 μM of piperine was used as the standard treatment level during infection and pretreatment of *H. pylori*. In both MOIs of 200 and 100, bacterial adhesion to the AGS cells was reduced up to 40% percent due to piperine treatment during infection and up to 25% in bacteria pretreated with piperine (Figure 2B), as determined by urease assay. Statistical analysis revealed that the piperine treatment during infection led to inhibition of bacterial adhesion (p < 0.01) and inhibition of piperine-pretreated *H. pylori* adhesion (p < 0.05).

### Effect of piperine on adhesin and flagellar molecules expression

**Figure 4:**
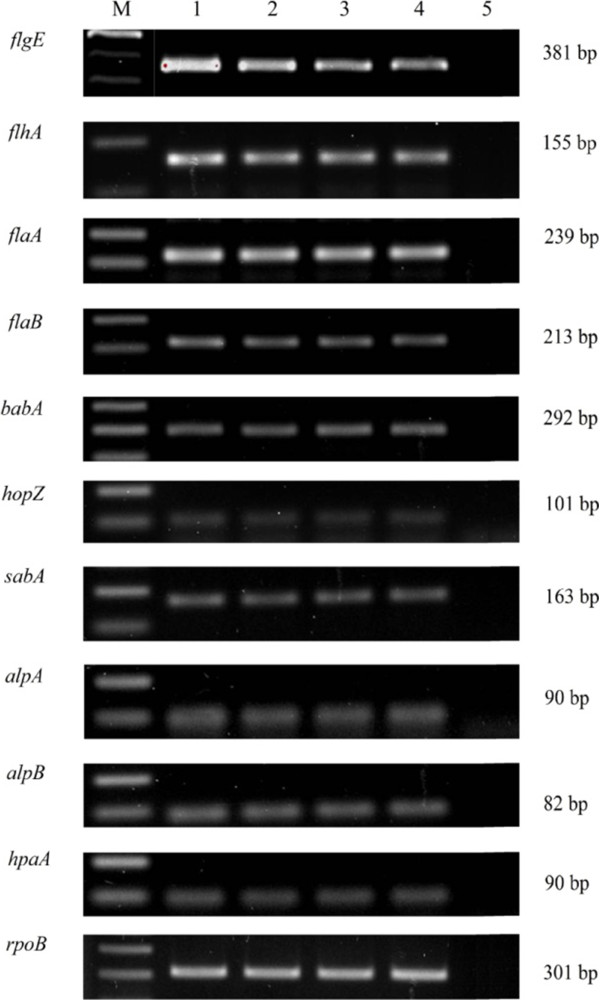
**Effect of piperine on** ***H. pylori*** **60190 adhesin and flagellar molecules.** *H. pylori* was treated with various concentrations of piperine and expression of adhesion and flagellar genes analyzed by reverse transcriptase PCR. M; Marker, 1; *H. pylori* – vehicle control, 2; *H. pylori* treated with 50 μM of piperine, 3; *H. pylori* treated with 75 μM of piperine, 4; *H. pylori* treated with 100 μM of piperine, 5; Negative control. An integral component of the flagellar export apparatus encoding gene *flhA* and flagellar hook component encoding gene *flgE* expression was diminished in piperine treatment in a dose dependent manner. The *rpoB* (encodes the β-subunit of RNA polymerase) was used as an internal control. The image is representative of three independent experiments.

*H. pylori* adhesion to the gastric epithelial is mediated by the adhesin molecules and the motility of the bacterium is driven by the flagella. As demonstated in the adhesion assay, *H. pylori* adhesion to gastric epithelial cells was inhibited significantly by the action of piperine. Therefore we performed RT-PCR to determine whether expression of the adhesin and flagellar genes was influenced by piperine. We tested the expression of adhesin genes *hopZ*, *babA*, *sabA*, *hpaA*, *alpA*, *alpB*, and flagellar genes *flhA*, *flaA*, *flaB* and *flgE*. Among these genes, flagellar integral membrane component encoding gene *flhA* and flagellar hook component encoding gene *flgE* expression was decreased dose dependently (Figure 4) in the bacteria treated with piperine, but the expression level of other molecules remained unchanged.

### Effect of piperine on *H. pylori*motility

**Figure 5:**
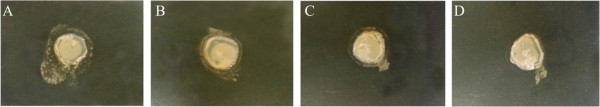
**Motility inhibition of piperine on** ***H. pylori*** **60190. A)** DMSO control; **B)** 50 μM piperine; **C)** 75 μM piperine; **D)** 100 μM piperine. Agar grown *H. pylori* was inoculated on motility medium and after three days the motility inhibition was observed and photographed. The piperine treatment reduced motility at 100 μM of piperine.

A motility agar containing 0.4% agar was used to determine the motility. *H. pylori* motility was abated in the medium, containing piperine compared to the DMSO containing control medium (Figure 5A). The swarming movement of the *H. pylori* decreased gradually at concentrations from 50 μM and declined at a concentration of 100 μM (Figure 5B-D).

### Protein levels of FlgE and FlhA

**Figure 6:**
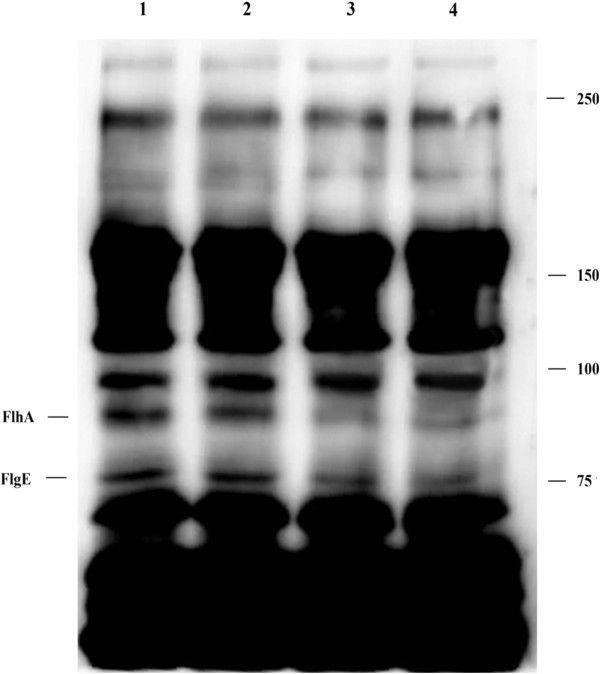
**Immunoblotting of** ***H. pylori*** **60190 whole cell antigen.** *H. pylori* was treated with various concentrations of piperine and protein levels of flagellar molecules were analyzed by immunoblotting. 1; *H. pylori* – vehicle control, 2; *H. pylori* treated with 50 μM of piperine, 3; *H. pylori* treated with 75 μM of piperine, 4; *H. pylori* treated with 100 μM of piperine. Flagellar hook component protein FlgE (77 KDa) and an integral component of the flagellar export apparatus FlhA (81 KDa) was separated by 7.5% SDS-PAGE and expression were diminished in piperine treatment in a dose dependent manner. Other proteins remained unchanged. The representative of three independent experiments.

RT-PCR results revealed that transcription of *flgE* and *flhA* was reduced by piperine treatment. To confirm this finding, we performed immunoblotting to determine whether the protein level of both protein molecules was diminished. Due to lack of specific antibodies to FlgE and FlhA, we used polyclonal rabbit anti- *H. pylori* whole cell antibody which we produced in our lab as described previously by Kim SH et al. [13]. The molecular weights of the *H. pylori* FlgE and FlhA are 77 KDa and 81 KDa, respectively as determined from the Helicobase [14] - a specific database for *H. pylori* nucleic acid and protein sequences. Based on the predicted molecular weight, we observed the suppression of both FlgE and FlhA protein synthesis in piperine treated cells (Figure 6). Expression of other proteins was unchanged in both piperine treated and untreated normal control.

## Discussion

In this study, we demonstrated the inhibitory effect of piperine on *H. pylori* growth and adhesion in gastric epithelial cells, which is the critical first step in the infection process. *H. pylori* is reported to be an organism whose drug resistance can increase over time [15]. At present, researchers are searching for bioactive compounds to treat bacterial infections due to an increase in drug resistance. Numerous reports have been published on various natural compounds and food supplements that inhibit *H. pylori* growth [16], adhesion [17] or toxin secretion [13]. The aim of our study was to explore an existing natural compound, which may act against *H. pylori*. Piperine has a known protective action against gastric ulcers [18]. It was observed that piperine inhibited *H. pylori* growth completely at concentrations of 125 and 250 μM. It was observed that 125 μM is the minimal inhibitory concentration of piperine on *H. pylori* growth and it is recommended to determine the half maximal inhibitory concentration (IC _50_) of piperine on *H. pylori*. Based on the standard recommendations for the IC_50_ experiment, we observed that the 115 μM of piperine was lethal to about 50% of cells. IC_50_ value was determined by the percentage of growth inhibition was plotted in a nonlinear regression curve. Results from both experiments shows that 125 μM of piperine completely inhibited the *H. pylori* where as 115 μM of piperine killed 50% of cells. Therefore, we used a 100 μM concentration of piperine for subsequent experiments to uncover the inhibition mechanisms, such as adhesion and secretion of toxins. In this study, our data clearly shows that piperine inhibited *H. pylori* adhesion to gastric carcinoma cells. When 100 μM of piperine was applied to *H. pylori*, attachment was reduced by up to 40%. It was assumed that a piperine might obstruct *H. pylori* adhesion by blocking adhesion molecules such as, AlpA-B, BabA, SabA, HopZ, HpaA and Lewis^x^-LPS, which would then lead to less adhesion to AGS cells compared to untreated bacteria. Another possibility is that piperine may influence the membrane proteins of AGS cells, which are the critical targets for *H. pylori* adhesion. Similarly, there was significant reduction in adhesion when organisms were pretreated with piperine for 24 hours, but the adhesion was slightly higher than that of piperine treatment during the infection. We assumed that the organism might be in an inactive state at sub-MIC level, but when it encountered fresh medium after the piperine treatment, it became more adherent than when piperine was administered during infection, but less than that of untreated control.

*H. pylori* adhesion inhibition by the action of piperine was determined by adhesion assay and this inhibitory mechanism was assessed by the RT-PCR, immunoblotting analysis and motility test. Six adhesion molecules and four flagellar molecules were tested to determine if the piperine had an influence on its expression. Expression of the six adhesion molecules *H. pylori* adhesin molecules were unchanged during the piperine treatment, but the expression of two flagellar molecules *flhA* and *flgE* were found to be decreased (Figure 4) in a dose dependent manner. The biosynthetic molecule *flhA*, which is the positive regulator for the various molecules, particularly *flaA* and *flaB* and these genes, encodes flagellin, which is the subunit of the filament part of the flagella [6]. Though there was a decrease in *flhA* expression, we found no impairment in *flaA* and *flaB* expression. However, we observed decrease in the expression of *flgE*, which encodes the subunit of the hook component of the flagella. Results from the previous experiments showed that the reduced adhesion and expression of flagellar molecules such as *flhA* and *flgE*. RT-PCR data revealed that the suppression of transcription in *flgE* and *flhA* due to the action of piperine in a dose dependent manner. To confirm this finding, we analyzed the expression of protein by immunoblotting. Immunoblot data also clearly showed suppression of both proteins due to piperine. Results from both experiments clearly show that piperine influences *flgE* and *flhA* at the RNA and protein level, which leads to a less degree of infection. To confirm that the decrease in the expression of flagellar molecules by piperine exerted an effect, motility test was performed and the motility of the *H. pylori* 60190 was decreased dose dependently (Figure 5). It has been reported that *H. pylori* flagella is not involved in the bacterial adhesion, however the genes involved in the flagellar biosynthesis regulation also take part in regulating the production of adhesion molecules and may regulate the bacterial adhesion [19]. Kao et al. [5], described that bacterial motility enhances the bacterial adhesion and is a good target for the control of bacterial colonization. Collectively, these results suggest that due to the suppression of the biosynthetic regulator gene *flhA* (integral membrane component of the export apparatus) and flagellar hook gene *flgE* because of piperine treatment may lead to the reduction in motility confirmed by the motility test. Due to decreased motility of *H. pylori*, the organism may be less attracted towards gastric epithelial cells, which results in the less adhesion compared with the untreated bacteria. As stated previously, piperine has been reported to have anticancer activities [10, 12]. We believe that daily consumption of black pepper, which is comprised of 5–9% piperine [20], reduces one’s chance of infection and of developing gastric cancer caused by *H. pylori*. This report provides us a clue that impairment in the flagellar export system may lead to the defects in the plausible toxic protein injection and further detailed studies are in progress to better understand the complete mechanisms behind the inhibition of *H. pylori* adhesion to gastric epithelial cells followed by colonization and the action against *H. pylori*-initiated oncogenesis by piperine.

## Conclusions

In summary piperine inhibits *H. pylori* growth, and adhesion. Due to suppression in the *flhA* and *flgE* expression leads to decrease in motility. To the best of our knowledge, this is the first report showing the inhibition of *H. pylori* growth and adhesion to gastric adenocarcinoma cells by a single compound, piperine. Further studies are needed to analyze the reason behind the suppression of flagellar gene expression.

## Materials and methods

### Bacterial strains and mammalian cell culture

*H. pylori* reference strains 60190, NCTC 11637, Tx30a were purchased from American Type Cell Collection (ATCC, Manassas, VA, USA). Strains 60190 (ATCC 49503) and NTCC 11637 (ATCC 43504) express an intact and functional cagPaI and possess an s1/m1 vacA toxin, whereas the strain Tx30a (ATCC 51932) expresses s2m2 vacA toxin, but does not possess the cagPaI. Bacteria were cultured on Brucella agar (Becton Dickinson, Braintree, MA, USA) supplemented with 10% fetal bovine serum (FBS; Gibco, Long Island, NY, USA) and *H. pylori* selective supplement (vancomycin – 10.0 mg/l, cefsulodin – 5.0 mg/l, trimethoprim – 5.0 mg/l, amphotericin B – 5.0 mg/l) (Oxoid, Hampshire, England) in humidified incubators at 37°C under an atmosphere of 5% CO_2_. AGS (gastric adenocarcinoma cell line) was purchased from the Korea Cell Line Bank (KCLB, Seoul, Korea) and cultured in Dulbecco’s Modified Eagle’s Medium (DMEM; Gibco) supplemented with 10% FBS and 100 μg/ml streptomycin, 100 U/ml penicillin (Gibco).

### Growth measurement

The effect of piperine on *H. pylori* growth was determined by a broth macrodilution method [21] and the *H. pylori* bacterial inoculum was equipped with a turbidity comparable to that of a 0.5 McFarland standard (1 × 10^8^ cells) in Mueller Hinton broth (Becton Dickinson) supplemented with 10% FBS. A stock solution of 200 mM piperine (Sigma Aldrich, St. Louis, MO, USA) was dissolved in dimethyl sulfoxide (DMSO, Sigma Aldrich) and filter-sterilized using a 0.20-μm syringe filter. The working concentration was added directly to the broth to obtain a desired final concentration of the piperine (250 μM). Subsequently, the broth was subjected to twofold serial dilution from 250 μM to 3.9 μM and incubated for three days under microaerophillic conditions. Vehicle control was maintained by adding equal concentrations of DMSO (alone) to a tube that contained the same number of cells as the experimental tubes, and did not show any inhibitory action on bacterial growth. After three days, the growth inhibition of piperine on *H. pylori* was determined by the optical density at 600 nm using a NanoQuant spectrophotometer (Infinite M200, (Tecan Austria GmbH, Grödig, Austria).

### Half maximal inhibitory concentration (IC_50_)

*H. pylori* 60190 was used in this study. Agar grown *H. pylori* was suspended in Mueller Hinton broth supplemented with 10% FBS with a turbidity comparable to that of 0.5 McFarland standard (1 × 10^8^ cells). The concentration of piperine was incorporated between 50 to 150 μM respectively. Incubation, optical density was followed as described in the growth measurement and the data was analyzed by GraphPad-Prism Analysis software (GraphPad-Prism Software Inc., San Diego, CA).

### Adhesion assay

An adhesion test was performed as described by Rokka et al. [22], and for the infection studies, AGS cells were resuspended in DMEM and 1 × 10^4^/100 μl cells were seeded in 96-well microtiter plates (Becton Dickinson) to form a confluent monolayer. *H. pylori* bacteria were harvested from three-day-old cultures on solid media and resuspended in PBS (Gibco). *H. pylori* bacteria were resuspended in antibiotic and serum free DMEM, which infected the monolayer that had been treated with various doses of piperine or pretreated with the sub-minimal inhibitory concentration of piperine for 24 hours. Pretreatment of *H. pylori* was equipped by agar grown bacteria suspended in sterile PBS and then resuspended in Brucella broth with 10% FBS at a turbidity value comparable to that of a 1.0 McFarland standard. Bacteria were then treated with 100 μM of piperine for 24 hours before the cells were collected and resuspended in FBS and antibiotic free DMEM and used for infection. In this adhesion assay, multiplicity of infection (MOI) ratios of 1:200 and 1:100 were used to infect the AGS cells. After 2 hours of infection, the monolayer was washed three times with PBS to remove residual and unattached bacteria. A urease test was performed to analyze the quantity of bacteria that had adhered to the surface of the AGS cells. The urease test was carried out by adding 100 μL of urease test solution (7 mM phosphate buffer pH 6.8, 110 mM urea, 10 mg/L phenol red) into each well of microtiter plate. After a reaction time of 60 minutes, absorbance values at 540 nm were recorded with a NanoQuant spectrophotometer. The relative adhesion level was calculated by dividing the absorbance value of the sample with the absorbance value of a DMSO control containing the same amount of *Helicobacter*.

### Reverse transcriptase polymerase chain reaction

*H. pylori* 60190 bacterial inoculum was equipped with a turbidity comparable to that of a 0.5 McFarland standard (1 × 10^8^ cells/ml) was grown in Brucella broth supplemented with 10% FBS in the presence or absence of piperine for three days at 37°C on a humidity chamber containing CO_2_ incubator. Various concentrations of piperine from 50, 75 and 100 μm were treated to the bacteria, respectively and vehicle control was also maintained. Total RNA was extracted using TRIzol (Invitrogen, Carlsbad, CA, USA) and RNA concentration was determined by NanoQuant spectrophotometer. Expression of *H. pylori* adhesion molecules, flagellar molecules and *rpoB* (internal control) was determined by RT PCR. Two microgram of total RNA was mixed with 0. 25 ng of random hexamers (Invitrogen) and cDNA was synthesized using 200 U of MMLV-RT (Invitrogen). PCR reactions were performed in a total volume of 20 μl consisting of 2 μl of 1/10 diluted cDNA, 2 μl of 10X PCR buffer (Tris–HCl [pH 9.0], 20 mM MgCl_2_, [NH_4_]_2_SO_4_), 2.5 mM dNTPs, 20 pmole of each primer, and 0.5 U G-Taq DNA polymerase (Cosmo Genetech, Seoul, Korea). PCR amplifications were carried out on a PTC-200 Peltier Thermal Cycler (BioRad, Toronto, Ontario) and PCR products were analyzed by electrophoresis on a 2.0% agarose gel containing 0.5 μg/ml of ethidium bromide. Gel images were captured and analyzed using the Quantity One System (Bio-Rad, Hercules, USA). The primer sequences and PCR conditions are listed in Table 1.

**Table 1:** **Primers used in this study**

Primer	Relevant sequence	Size of amplicon	Amplification cycles	Annealing temperature	References
*flgE*- F	5′ CCGATCAAATCCTTAACACC 3′	381	30	52	This study
*flgE*- R	3′ AGGCTTAAAAACATGCGAAC 5′				
*flhA*- F	5′ TCATTGGAGGGTTTTTAGTGG 3′	155	26	60	[23]
*flhA*- R	3′ GGTGCGAGTGGCGACAAT 5′				
*flaA*- F	5′ TAGACACCACCAACGCTAAA 3′	239	28	62	This study
*flaA*- R	3′ TGCATTCTAGGGGGTTGTAT 5′				
*flaB*- F	5′ GTCAATGGCGTGAATGATTA 3′	213	30	60	This study
*flaB*- R	3′ ATTCACGGTCCCAATTTCTA 5′				
*babA*- F	5′ ATCGATCCACTTCCATCACT 3′	292	40	48	This study
*babA*- R	3′ GTTACGCTTTTGCCGTCTAT 5′				
*hopZ*- F	5′ GCGCCGTTACTAGCATGATCA 3′	101	24	60	[24]
*hopZ*- R	3′ GAAATCTTTCGGCGCGTTT 5′				
*sabA*- F	5′ AAAGCATTCAAAACGCCAAC 3′	163	24	60	This study
*sabA*- R	3′ CCCGCATAAAGACTCCAAAA 5′				
*alpA*- F	5′ GCACGATCGGTAGCCAGACT 3′	90	22	60	[24]
*alpA*- R	3′ ACACATTCCCCGCATTCAAG 5′				
*alpB*- F	5′ ACGCTAAGAAACAGCCCTCAAC 3′	82	26	60	[24]
*alpB*- R	3′ TCATGCGTAACCCCACATCA 5′				
*hpaA*- F	5′ GAGCGTGGTGGCTTTGTTAGT 3′	90	26	60	[24]
*hpaA*- R	3′ TCGCTAGCTGGATGGTAATTCA 3′				
*rpoB*- F	5′ TTTAGGTAAGCGCGTGGATT 3′	301	27	59	This study
*rpoB*- R	3′ AATCAGCTTTGGATGGAACG 5′				

### Motility test

Motility testing was performed as described by Suerbaum et al. [25]. In brief, Brucella agar supplemented with 10% BSA (bovine serum albumin, Gibco) was used and two layered plates were prepared to determine the motility. Bottom layer contained 1.5% agar, which poured on the plates followed by the soft upper layer contained 0.4% agar and piperine (50, 75, and 100 μM). Small slices of Brucella agar, which contained densely grown *H. pylori* 60190 was inoculated by grown side of the agar slice facing the soft layer and incubated for three days under microaerobic condition [25].

### Immunoblotting

*H. pylori* 60190 was used in this study. *H. pylori* treatment was followed as described before in RT- PCR. Cells were harvested then washed with sterile PBS and lysed by RIPA buffer (Millipore, Billerica, MA, USA) for 30 minutes on ice bath and sonicated for 2 minutes with 10 second intervals (Sonicator XL-2020, Heat Systems Ultrasonics, Pittsburgh, PA, USA) followed by centrifugation on 14, 000 RPM for 15 minutes at 4°C. Protein concentrations were determined by Lowri’s method using NanoQuant spectrophotometer. Proteins were separated by sodium dodecyl sulfate polyacrylamide gel electrophoresis (SDS-PAGE) and transferred to BioTrace nitrocellulose membranes (Pall Corporation, Ann Arbor, MI, USA). Membranes were then blocked by 5% skim milk dissolved in PBS with 0.025% Tween 20 (PBST) then probed overnight with polyclonal rabbit anti- *H. pylori* whole cell antibody [13]. After washing with PBST, bound antibody was detected with a horseradish peroxidase conjugated anti-rabbit IgG secondary antibody (Santa Cruz Biotechnology, CA, USA). The blots were then developed by using EZ- Western Lumi Femto (Daeil lab service, Seoul, South Korea) and the signals were detected using a Fusion Solo Detector (Vilber Lourmat, Marne La Vallee, France).

### Statistical analysis

Data were analyzed using the Student’s *t*-test and expressed as mean values of at least three independent replications. Differences were considered to be highly statistically significant when p <0.01 and significant at p <0.05. The mean value of three independent experiments were analysed for IC_50_ was calculated by nonlinear regression from logarithmic concentrations of piperine Vs normalized response with variable slope using Graphpad Prism.

## Abbreviations

*H. pylori*: *Helicobacter pylori*
alpA: alpB: Adherence-associated lipoprotein A and B
babA: Blood group antigen-binding adhesion
sabA: Sialic acid-binding adhesion
hopZ: *Helicobacter pylori* outer membrane protein
hpaA: *Helicobacter pylori* adhesin A
flhA: Flagellar biosynthesis protein
flgE: Flagellar hook protein
flaA: Flab: Flagellin A and B
RT-PCR: Reverse transcriptase-polymerase chain reaction
sub-MIC: Subminimal inhibitory concentrations
DMSO: Dimethyl sulfoxide
DMEM: Dulbecco’s modified eagle medium
FBS: Fetal bovine serum
BSA: Bovine serum albumin
MMLV-RT: Moloney murine leukemia virus- reverse transcriptase
IC_50_: Half maximal inhibitory concentration
SDS-PAGE: Sodium dodecyl sulfate polyacrylamide gel electrophoresis
PBST: PBS with 0.025% Tween 20
RIPA buffer: Radio-immunoprecipitation assay buffer.
